# Embedded monitoring system and teaching of artificial intelligence online drug component recognition

**DOI:** 10.1515/biol-2022-0795

**Published:** 2024-06-11

**Authors:** Li Ding, Zhengrong Wu, Junmin Zhang, Quanyi Zhao, Xiaoling Chen, Zhong Jia, Dian He

**Affiliations:** Materia Medica Development Group, Institute of Medicinal Chemistry, Lanzhou University School of Pharmacy, Lanzhou 730000, China; The No. 2 People’s Hospital of Lanzhou, Lanzhou 730000, China

**Keywords:** drug composition identification, embedded monitoring system, drug teaching, artificial intelligence

## Abstract

Drug testing has many test elements. It aims to prevent unqualified drugs from entering the market and ensure drug safety. The existing artificial intelligence (AI) online monitoring system identifies active ingredients in the process of use. Owing to their openness, data are easy to be lost, failing to meet user needs and inducing a specific impact on the use of the monitoring system. With the continuous development of computer and measurement technologies, various biochemical data are increasing at an unprecedented speed, and numerous databases are emerging. Extracting patterns from considerable known data and experimental facts is an essential task for a wide range of biological and chemical workers. Pattern recognition is one of the essential technologies for data mining. It is widely used in industry, agriculture, national defense, biomedicine, meteorology, astronomy, and other fields. To improve the effect of the online drug ingredient recognition system, this study used AI to design an online drug ingredient recognition-embedded monitoring system and applied AI to the teaching field to improve teaching efficiency. First, this study constructed the framework of the AI online drug ingredient recognition-embedded monitoring system and introduced the process of online drug ingredient recognition. Then, it introduced the pattern recognition method, constructed the pattern recognition system, and presented the pattern recognition algorithm and the algorithm evaluation index. Afterward, it used pattern recognition to conduct a qualitative analysis of the infrared spectrum of drug components and introduced the overall process of the qualitative analysis. In addition, this study employed AI to implement changes to the embedded system instruction in colleges and universities, summarizing the current issues. The impact of drug component recognition and the educational impact of embedded systems were investigated in the experimental portion. The experimental findings demonstrated the excellent accuracy, sensitivity, specificity, and Matthew correlation coefficient of the online drug component recognition-integrated monitoring system in this work. Compared with that of other systems, its average drug component recognition accuracy was above 0.85. Students in five majors reported high levels of satisfaction with the embedded system teaching, which is better for delivering college instruction.

## 1 Introduction

With the rapid development of modern spectral analysis technology, the analysis and identification of effective components have become more practical than before. Present drug analysis technology mainly includes microbiology, high-performance liquid chromatography, and infrared spectroscopy. The microbiological method is to use the inhibition of drugs on the growth of microorganisms. High-performance liquid chromatography is to transport a specific liquid dissolved in a test substance to a chromatographic column for separation and measurement through a high-pressure transmission system. Infrared spectroscopy is a widely used analytical method that analyzes substances by measuring the interaction between substances and spectra.

Many studies have been conducted on drug recognition. Khan et al. adopted a drug reuse method to identify 3C-like protease and 2′-*O*-ribomethyltransferase inhibitors [1]. Chang et al. used single-cell RNA sequencing to track differential cloning reactions [2]. Islam et al. performed molecular simulation to identify the best candidate for antiviral phytochemicals that can be used as effective inhibitors of major protease [3]. Drug identification is an essential step in drug research, but studies on drug identification are not deep enough and need to be further strengthened.

Artificial intelligence (AI) has been applied in drug research. Zeng et al. provided a powerful network-based deep learning method for target recognition to accelerate drug reuse [4]. Zhao et al. used convolutional and deep neural networks to identify drug target interaction [5]. Kobylka et al. outlined the molecular basis of drug inhibitor binding, including insights into the mechanism of AcrB in drug efflux. They also summarized mutation analysis of the acrB gene and reported the impact of each substitution on antibiotic resistance in *Escherichia coli* [6]. Gupta et al. studied the application of machine and deep learning algorithms in drug design and development [7]. Magge et al. evaluated the role of the deep learning pharmacovigilance pipeline in adverse drug events [8]. Dara et al. reviewed the application of machine learning tools and technologies in drug discovery [9]. Chandrasekaran et al. studied the role of image analysis in drug discovery [10]. Wang applied computer-aided drug design technology to identify promising drug reuse candidates quickly [11]. Monteiro et al. examined how drugs can be used in animal clinical trials to successfully identify and treat pain in animals [12]. However, these scholars did not consider privacy and security issues, as well as the excessive reliance on AI, in drug testing. The system designed in this study encrypts data to prevent data leakage, and automatic analysis technology for the chemical composition of drugs is added to the system. AI systems are regularly evaluated and validated to ensure their accuracy and reliability.

**Figure 1:**
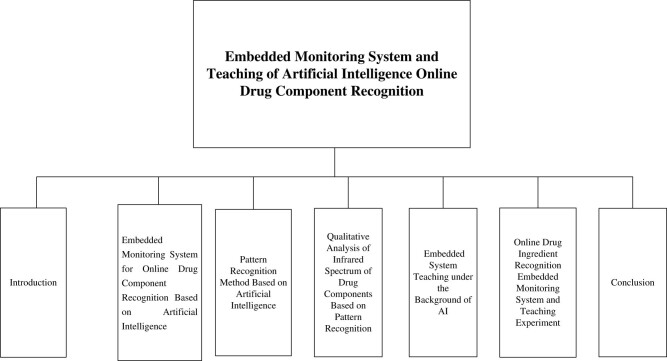
Overall structure of this study.

To improve the effectiveness of online drug component recognition systems, this study used AI to conduct online drug component recognition research. First, this study constructed an online drug component recognition-embedded monitoring system and a pattern recognition system. Then, it used the pattern recognition method to analyze the infrared spectrum of drug components qualitatively. Finally, this work introduced the shortcomings and improvement methods of embedded system teaching under the background of AI. The overall structure of this study is shown in Figure 1.

## 2 Embedded monitoring system for online drug component recognition based on AI

**Figure 2:**
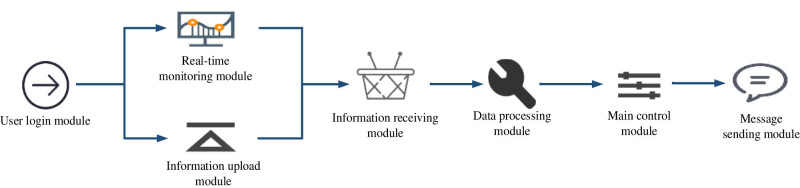
AI online drug ingredient recognition-embedded monitoring system.

The embedded monitoring system of AI online drug ingredient recognition is shown in Figure 2.

The monitoring system consists of the following modules: user connection, information upload, real-time monitoring, data receiving, data processing, general control, and information transmission. After detecting the user information, the user connection module activates the connection. With the help of the information download module, users can download standard drug information. Information on the real-time monitoring of drugs is gathered using this module. The data receiving module is used to receive the standard information about the drug that was downloaded from the information download module and the drug information from the real-time monitoring module. It instantly transmits the usual drug information that was received by the data-processing module.

## 3 Pattern recognition method based on AI

### 3.1 Pattern recognition system

**Figure 3:**
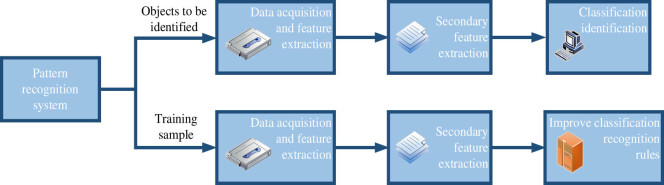
Principle of the pattern recognition system and recognition process.

Pattern recognition is based on the attributes of the object to be tested centered on the computer system. It uses a specific analysis algorithm to define the category, which can display the classification and identification results as accurately as possible. Its composition and environment determine the characteristics of matter, and the understanding of things depends on the understanding of such characteristics. The characteristics of things with diverse properties differ in quantity and quality. Therefore, as long as people find the properties related to the properties of things, they can classify things with different properties. Then, things with unknown attributes can be distinguished to predict their attributes [13]. The principle of the pattern recognition system and recognition process is shown in Figure 3.

In small spaces, such as 2D or 3D, pattern recognition can be used for all recognition tasks, such as face recognition and speech recognition. The measurements obtained with modern analyzers are high-dimensional and contain much relevant information. People need help to intuitively access pattern information, which requires the assistance of mathematics and computer technology. Although the level of machine recognition remains considerably behind the human brain, with the development of pattern recognition theory and other related disciplines, its functions would be more powerful and extensive.

Feature extraction is an essential stage of pattern recognition. It has been widely studied but without universal theory to follow. Generally, it should select the features suitable for classification in accordance with the research issues and topics, combined with experience. The principle of feature extraction is to obtain good classification results. Sometimes, when meeting the requirements of classification and recognition accuracy, it can select features that play an essential role in correct classification and recognition in accordance with specific criteria and try to complete classification and recognition tasks with fewer features [14].

Machines with classification and recognition functions must first input the classification and recognition knowledge of objects into the machine to create classification and recognition rules and classification processes. This step usually requires constant repetition to correct errors and improve defects, as well as change feature extraction methods, including feature selection schemes, decision rules, and parameter variations. Finally, the accuracy of system identification can be improved to meet the design requirements. At present, this machine learning process requires human intervention [15].

The classification rules and procedures generated during and after learning are used to detect unknown objects. The more classification and recognition knowledge and methods entered by the machine and the more understanding of relevant objects, the stronger the recognition ability of the system and the higher the recognition accuracy.

### 3.2 Pattern recognition algorithm

#### 3.2.1 Mode similarity measurement

In pattern recognition, the relationship between samples or variables is usually necessary to describe, and two basic methods can be used to describe these relationships. One is the similarity coefficient. Similar samples have high similarity, whereas heterogeneous samples have low similarity. Standard similarity measures are correlation coefficient and square cosine. The other is to test the relationship between samples or variables through the distance of sampling points in multidimensional space in accordance with the principle of similar clustering [16]. Given a specific type of substance to be measured, the substance must keep a small distance from the aggregation of such substances in the n-dimensional space and a considerable distance from the aggregation of substances that are not similar in the n-dimensional space. The mode similarity measurement is carried out using the angle similarity coefficient.

The angle similarity coefficient is as follows:

(1) \[{\mathrm{alsas}}{a}_{{ij}}=\frac{{\sum }_{k=1}^{n}{x}_{{ik}}{x}_{{jk}}}{\sqrt{\left(\phantom{\rule[-0.75em]{}{0ex}}{\sum }_{k=1}^{n}{x}_{{ik}}^{2}\right)\left(\phantom{\rule[-0.75em]{}{0ex}}{\sum }_{k=1}^{n}{x}_{{jk}}^{2}\right)}}.]\]

The smaller the included angle is, the more similar it is, similar to comparing two spectra. The two spectra are completely the same when \[\cos {a}_{{ij}}=1]\] , and they are completely different when \[\cos {a}_{{ij}}=0]\] .

#### 3.2.2 Feature extraction and optimization

Genetic algorithm is an effective nonlinear global optimization search method. It simulates the evolution process of natural organisms, randomly searches the optimization space, and generates global optimization. The numerical genetic algorithm sets the different parameters to be optimized as chromosomes. The parameters are all genes of chromosomes. In accordance with the adaptability of a chromosome to the environment, it can control its reproduction through various genetic operations, remove wrong parameters, and keep suitable parameters. The individuals that undergo genetic operation form a new population of the next generation. The next series of evolution of this new population ends after obtaining the best chromosome.

The basic process of genetic algorithm includes initialization, evaluation, selection, heredity (gene exchange), and population mutation. Two conditions are considered to terminate the process of the genetic variation cycle. One is that the solution is already the best solution to the problem or a satisfactory solution, and the other is the control of genetic algebra [17].

Genetic algorithm uses simple coding methods and genetic mechanism to represent complex phenomena so that very complex problems can be solved. In particular, because it is not restricted by the limited assumptions of the search blank problem, it is not necessary to comply with the assumptions of continuity, compatibility, and single mode. It has considerable application potential in chemistry.

#### 3.2.3 Classification recognition algorithm

Classification includes searching a group of models (or functions) in the learning data, describing the typical characteristics of the dataset to classify and identify the distribution or category of unknown data, and assigning unknown situations to discrete categories. Current classification methods include support vector machines and artificial neural networks.

Small-sample, nonlinear, and high-dimensional learning issues can be solved using support vector machines, a unique tool for implementing statistical learning theory that does not require enough data to create feature space. A classification interface can only be created with a minimal amount of examples.

The basic idea of vector machines is to first transform the input space into a high-dimensional feature space through nonlinear transformation and then find the best linear classification surface in the new space by defining the corresponding integral function. A hyperplane is defined between the midpoints of the feature space, and the kernel function *k* can subsequently be specified. This technology avoids the computational load that obviously represents the eigenvector. The discriminant functions of the support vector include the following:

(2) \[f\left(x)=\mathrm{sgn}\left\{\phantom{\rule[-0.95em]{}{0ex}}\mathop{\sum }\limits_{i=1}^{n}{\alpha }_{i}\left\times {y}_{i}K\left({x}_{i}{\mathrm{\cdot }}x)+{b}^{* }\right\}.]\]

Among them, \[({x}_{i}\left,{y}_{i})]\] is the sample set of linear segmentation, \[\mathrm{sgn}\left()]\] is the sign function, \[{\alpha }_{i}]\] is the Lagrange coefficient, and \[{b}^{* }]\] is the classification threshold.

### 3.3 Evaluation index of pattern recognition algorithm

The commonly used taxonomic performance indicators include accuracy (ACC), sensitivity (SEN), specificity (SPE), and Matthew correlation coefficient (MCC).

ACC only reflects the general predictive effect and does not describe the predictive effect of positive (or negative) samples [18]. Differences exist between the two. SEN and SPE can reflect the expected impact of positive and negative samples. However, a single SEN or SPE does not indicate the validity of the prediction, so the composite indicator MCC is usually used.

(3) \[{\mathrm{ACC}}=\frac{{\mathrm{TP}}+{\mathrm{TN}}}{{\mathrm{TP}}+{\mathrm{TN}}+{\mathrm{FP}}+{\mathrm{FN}}},]\]

(4) \[{\mathrm{SEN}}=\frac{{\mathrm{TP}}}{{\mathrm{TP}}+{\mathrm{FN}}},]\]

(5) \[{\mathrm{SPE}}=\frac{{\mathrm{TN}}}{{\mathrm{TN}}+{\mathrm{FP}}},]\]

(6) \[{\mathrm{MCC}}=\frac{{\mathrm{TP}}{\mathrm{\cdot }}{\mathrm{TN}}-{\mathrm{FN}}{\mathrm{\cdot }}{\mathrm{FP}}}{\sqrt{({\mathrm{TP}}\left+{\mathrm{FN}}\left){\mathrm{\cdot }}({\mathrm{TP}}\left+{\mathrm{FP}}\left){\mathrm{\cdot }}({\mathrm{TN}}\left+{\mathrm{FN}}\left){\mathrm{\cdot }}({\mathrm{TN}}\left+{\mathrm{FP}})}}.]\]

TP represents true positive, predicted positive, and actual positive; FP stands for false positive, the predicted value is positive, and the actual value is negative; FN stands for false negative, the predicted value is negative, and the actual value is positive; TN represents true negative, predicted negative, and actual negative.

## 4 Qualitative analysis of the infrared spectrum of drug components based on pattern recognition

### 4.1 Infrared spectrum steps for drug components

For the infrared spectrum data of drugs, the data refer to a fuzzy collection. The differences in the basic characteristics of samples cannot be identified by spectral data, and the samples to be identified are usually fuzzy sets in the application process. Therefore, the model is established in the spectral data of the samples with known attributes. Then, the approximate principle of fuzzy pattern recognition technology is used to calculate the classification of unknown patterns. Combined with fuzzy pattern recognition technology, the steps for qualitative analysis of the infrared spectrum of active components are as follows:

(1) The infrared spectrometer can be used to measure the spectral data in the sample to be measured, preprocess the spectral data, and eliminate the spectral noise.
(2) The original spectral data can be dimensioned down.
  Because excessive original spectral variables exist, the complexity of the problem analysis is undoubtedly increased, and a certain correlation occurs between multiple spectral variables, which often leads to low accuracy of the recognition model. When the original spectral data are used as the input data of fuzzy morphological recognition, the stability is reduced, and long-term modeling may lead to defects. Therefore, before the infrared spectrum detection model is created, the size of spectral variables should be reduced first. Before fuzzy pattern recognition technology is used to model, traditional principal component analysis is employed to reduce the dimension of the infrared spectrum [19]. The principal component analysis method allows a small number of new variables to be selected instead of the original spectral variables without losing the basic spectral information. In other words, the space of high-dimensional original spectral variables can be replaced with a low-dimensional spectral variable space to achieve the goal of reducing dimensions.
(3) New variables are used as indicators for each known category sample in the model, and statistical software is adopted to determine the maximum proximity of each known category and each category to be tested.
(4) To analyze the identification results, the abnormal data are cleared, and step 3 is repeated until the results are satisfactory.

### 4.2 Qualitative analysis of drug components by infrared spectroscopy

#### 4.2.1 Dimension reduction of spectral variables via principal component analysis

When analyzing data, complex datasets often need to be simplified. The *p*-measurement system composed of p indicators would be simplified to *m*(*m* < *p*) – measurement. Basic component analysis is a statistical analysis method that converts several indicators into several comprehensive indicators. In the process of studying variables, excessive variables often exist with a certain correlation between them. The measured data overlap with the information to a certain extent. If many variables are available, the sampling rules in the high-dimensional space are difficult to study. Principal component analysis uses the method of reducing measurement to identify multiple elements. Most of the information of these elements reflects the content of the original variables, and they are not related to each other to achieve the goal of simplification.

#### 4.2.2 Fuzzy pattern recognition of drug infrared spectrum

The point matrix of principal components is introduced into the recognition model. The Data Processing System (DPS) statistical analysis software is used to calculate the proximity of drug samples, followed by the category.

## 5 Embedded system teaching under the background of AI

### 5.1 Current challenges in embedded system teaching

Embedded systems are useful and place great emphasis on learning fundamental technological abilities and using technology in the teaching process. As a result, relevant research projects and competitions are covered in class and during the break. The mastery of theoretical information and the abilities used in practical competitions must be encouraged during the educational process. However, numerous universities still use traditional methods of instruction, which emphasize theory over practice almost exclusively. No real connection exists between theory and practice, which is primarily reflected in the following areas:

(1) Many theoretical courses but relatively few practical courses exist in the learning process.
(2) Although some colleges and universities pay considerable attention to practical teaching and increase the experimental time, the organization of the experimental content is unreasonable, the experimental task is extremely simple, and few comprehensive design experiments are available. The experimental class is only a course to complete the course tasks. Students only need to learn step by step and cannot use knowledge after learning.
(3) When selecting textbooks, colleges and universities mainly focus on the expression of theoretical knowledge. The practice part is relatively small, lacking examples and design tasks, which is not in line with the current goal of talent training in colleges and universities.
(4) Teachers lack of professional and technical abilities, product-related comprehensive project experience, and backward teaching methods; the teaching process is only theoretical explanation; and students lack learning interest and enthusiasm.
(5) The evaluation and promotion of teachers mainly depend on their work and scientific research. Hence, teachers spend most of their time on projects and work, and spending the rest of their time and energy on training talents is difficult.
(6) The existing courses are outdated and cannot keep up with the current pace of relevant industries and technologies.
(7) The enthusiasm of students to participate in various competitions is low, the number of students who can complete tasks and win prizes is small, and students lose the opportunity to exercise in competitions, which has a negative impact on the formation of applied talents.

### 5.2 Teaching reform of embedded system course under the background of AI

#### 5.2.1 Course reform of embedded software foundation

As one of the programming languages, C language is an entry-level language for software development and one of the leading development languages for embedded systems and AI technology. Therefore, the C programming course is particularly important for students studying embedded system programs.

For each programming language, data structure is the prerequisite for realizing software functions. Especially for the low- and middle-level embedded systems with insufficient internal resources, the running speed is relatively slow, and the various links of software design are closely related. They are small and simple data structures. The data structure of software is different from that of traditional computers.

Database is the core of AI and an important part of embedded systems. It is a useful tool for developing and managing user applications. Students have mastered database programming technology and can use capacitors to manage data in large embedded and artificial applications.

#### 5.2.2 Revising the training plan

Revising the curriculum, adjusting the time and proportion of the curriculum, and correspondingly increasing the proportion of comprehensive, creative, and open textbooks are necessary. In view of the rapid development of application technology, the teaching of application functions in talent development planning has been increased.

#### 5.2.3 Strengthening the development of practical learning platform

A comprehensive practical learning platform should be actively implemented, and the construction of infrastructure, such as competition bases, comprehensive learning centers, and innovation and entrepreneurship practice learning bases, should be strengthened. This article introduces relevant research projects for students to practice on the platform.

#### 5.2.4 Improving teaching methods and curriculum evaluation system

Practical training materials suitable for applied learning, including practical case studies and special training manuals for open experiments, can be chosen. The analysis of students’ current learning level and motivation, the improvement of the curriculum, and the introduction of effective learning methods are also possible. The project method is introduced into the curriculum to realize knowledge transfer and technology development, achieve comprehensive education objectives, guide and encourage students to apply for different types of innovative science and technology projects, and develop students’ research and technical skills.

To develop evaluation methods that can help students learn, the focus is to mobilize their enthusiasm and improve their practical and innovative skills. Some practical evaluations can be added to the traditional graduation evaluation mechanism to reduce the proportion of theoretical achievements and increase the proportion of practical achievements. This approach can stimulate students’ learning motivation and lay a good foundation for the development of applied engineering talents.

## 6 Online drug ingredient recognition-embedded monitoring system and teaching experiment

To identify the components of five drugs, the recognition method used is the online drug component recognition-embedded monitoring system proposed in this study and other drug component monitoring systems. The teaching effect of the embedded system under the background of AI is also studied.

### 6.1 Identification of drug components

**Figure 4:**
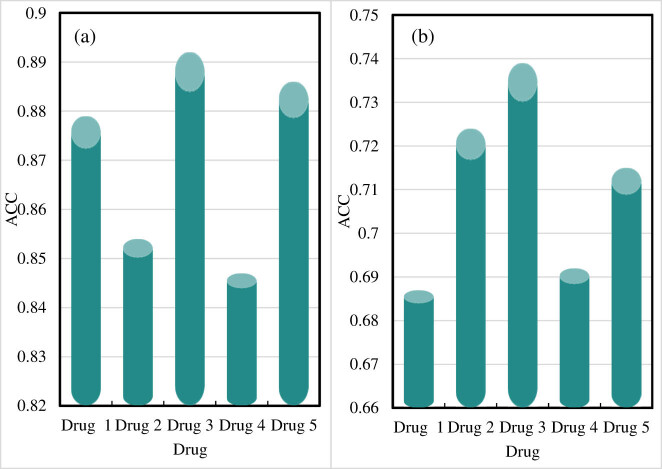
ACC of drug composition identification results. (a) ACC of the drug component identification results of the online drug component identification-embedded monitoring system proposed in this study. (b) ACC of the drug composition identification results from other drug composition monitoring systems.

The ACC of drug component identification results is shown in Figure 4.

Figure 4(a) shows the ACC of the online drug component identification-embedded monitoring system proposed in this study, and Figure 4(b) presents the ACC of other drug component monitoring systems.

When the online drug component identification-embedded monitoring system proposed in this study is used for drug component identification, the result for drug 1 (ACC) is 0.879. The ACC for drug 2 is 0.854, the ACC for drug 3 is 0.892, and the ACC for drug 4 is 0.847. The ACC for drug 5 is 0.886, and the average ACC of the online drug component identification-embedded monitoring system presented in this study is 0.872.

The ACC for drug 1 is 0.687, and the ACC for drug 2 is 0.724 when other drug component monitoring systems identify drug components. The component identification for drug 3 has an ACC of 0.739, and that for drug 4 has an ACC of 0.692. The ACC for drug 5 is 0.715, and the average ACC obtained by other drug component monitoring systems is 0.711.

**Figure 5:**
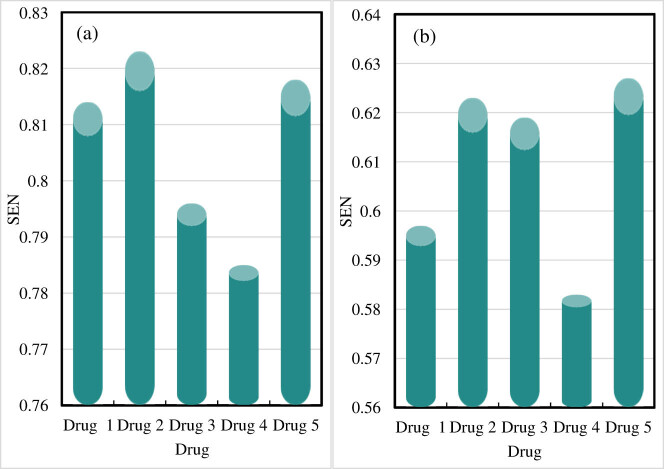
SEN of drug component identification results. (a) SEN of the drug component recognition results obtained by the online drug component recognition embedded monitoring system presented in this study. (b) SEN of the drug component identification results of other drug component monitoring systems.

The SEN of drug component identification results is shown in Figure 5.

Figure 5(a) shows the drug component identification result SEN of the online drug component identification-embedded monitoring system presented in this study, and Figure 5(b) demonstrates the drug component identification result SEN of other drug component monitoring systems.

For drug 1, when using the online drug component identification-embedded monitoring system proposed in this work, the SEN of the drug component identification result is 0.814; when using other drug component monitoring systems, the SEN of the drug component identification result is 0.597. For drug 2, the SEN of drug component identification is 0.823 when using the proposed system and 0.623 when using other systems. For drug 3, the SEN of the drug component identification result is 0.796 when using the proposed system and 0.619 when using other systems. For drug 4, the SEN is 0.785 when using the presented system and 0.583 when using other systems. For drug 5, the SEN is 0.818 when using the presented system and 0.627 when using other systems.

The average SEN of the online drug component recognition-embedded monitoring system proposed in this work is 0.807, and the average SEN of other drug component monitoring systems is 0.61.

**Figure 6:**
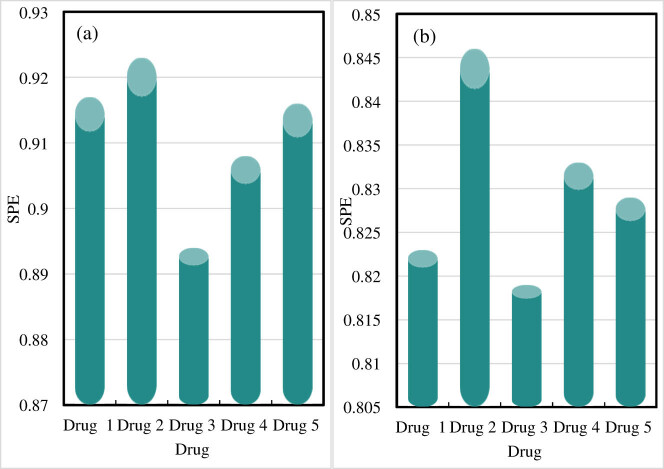
SPE of drug component identification results. (a) SPE of the drug component recognition results from the online drug component recognition-embedded monitoring system proposed in this work. (b) SPE of the drug component identification results of other drug component monitoring systems.

The SPE of drug component identification results is shown in Figure 6.

Figure 6(a) shows the SPE of the drug component identification results of the online drug component identification-embedded monitoring system proposed in this work, and Figure 6(b) depicts the SPE of the drug component identification results of other drug component monitoring systems.

The SPE of the drug component identification results for drugs 1 and 2 are 0.917 and 0.923, respectively, when the online drug component identification-embedded monitoring system in this work performs drug component identification. The component identification for drug 3 has an SPE of 0.894, and that for drug 4 has an SPE of 0.908. The SPE for drug 5 is 0.916, and the average SPE using the proposed system is 0.912.

When other drug component monitoring systems identify drug components, the SPE for drug 1 is 0.823, and the SPE for drug 2 is 0.846. The SPE for drug 3 is 0.819, and the SPE for drug 4 is 0.833. The SPE for drug 5 is 0.829, and the average SPE of the drug component identification results of other systems is 0.83.

**Figure 7:**
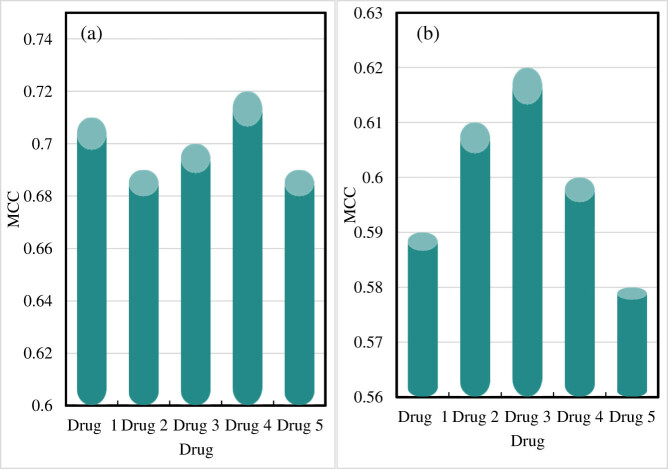
MCC of drug component identification results. (a) MCC of the drug component recognition result obtained by the online drug component recognition-embedded monitoring system in this work. (b) MCC of the drug component identification results from other drug component monitoring systems.

The MCC of drug component identification results is shown in Figure 7.

Figure 7(a) shows the MCC of the online drug component identification-embedded monitoring system in this work, and Figure 7(b) indicates the MCC using other drug component monitoring systems.

The drug component recognition result for drug 1 is 0.71 when the online drug component recognition-embedded monitoring system in this study performs drug component recognition. For drugs 2 and 3, the MCCs of drug component identification are 0.69 and 0.7, respectively. The MCC for drug 4 is 0.72. The component identification result for drug 5 has an MCC of 0.69, and the average MCC using the proposed system is 0.702.

When other drug component monitoring systems perform drug component identification, the MCC for drug 1 is 0.59, and that for drug 2 is 0.61. The MCC for drug 3 is 0.62, that for drug 4 is 0.6, and that for drug 5 is 0.58. The average MCC of the drug component identification results of other systems is 0.6.

The comprehensive results of drug component identification are shown in Table 1.

**Table 1:** Comprehensive results of drug component identification

Target	Proposed system	Other systems
ACC	0.872	0.711
SEN	0.807	0.61
SPE	0.912	0.83
MCC	0.702	0.6

The comparative data indicate that the ACC, SEN, SPE, and MCC of the drug component identification system in this study are higher than those of other systems. The average accuracy of the presented system is above 0.85, which is a high value.

### 6.2 Embedded system teaching under the background of AI

**Figure 8:**
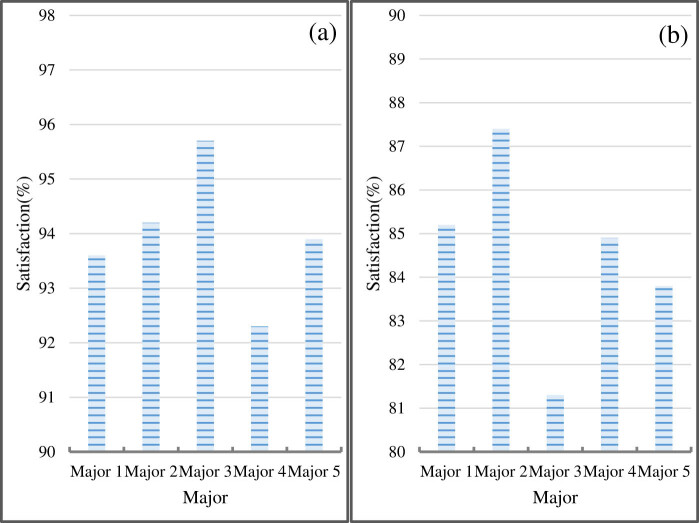
Comparison of teaching satisfaction. (a) Students’ satisfaction with embedded system teaching. (b) Students’ satisfaction with traditional teaching.

This study surveys the students of five majors in a university and compares their satisfaction with embedded system teaching and traditional teaching. The results are shown in Figure 8.

Figure 8(a) shows students’ satisfaction with embedded system teaching, and Figure 8(b) illustrates students’ satisfaction with traditional teaching.

Students in major 1 are 93.6% satisfied with embedded system teaching and 85.2% satisfied with traditional teaching. Students in Major 2 are 94.2% satisfied with embedded system teaching and 87.4% satisfied with traditional teaching. Students in Major 3 are 95.7% satisfied with embedded system teaching and 81.3% satisfied with traditional teaching. Students in major 4 are 92.3% satisfied with embedded system teaching and 84.9% satisfied with traditional teaching. Students in Major 5 are 93.9% satisfied with embedded system teaching and 83.8% satisfied with traditional teaching.

In comparison with traditional teaching, which has an average student satisfaction rating of 84.5, embedded system teaching has a rating of 93.9. More than 90% of the students in the five majors are satisfied with embedded system teaching, which is greater than the percentage of students who are satisfied with traditional teaching. This finding suggests that embedded system teaching is more effective and can pique students’ interest in learning.

## 7 Conclusion

In this study, AI was used to assess the online drug ingredient recognition-embedded monitoring system, and the teaching mode under the background of AI was discussed. This work constructed an embedded monitoring system framework for online drug component identification and selected pattern recognition methods for drug component identification. It introduced the measurement of pattern similarity, feature extraction and optimization, and classification recognition algorithm and selected ACC, SEN, SPE, and MCC as the evaluation indicators for pattern recognition algorithm. Pattern recognition was used to qualitatively analyze the infrared spectrum of drug components, and the original spectral data were preprocessed and dimensionally reduced. The DPS statistical analysis software was utilized to calculate the proximity of drug samples and determine their category. Then, this study analyzed the problems existing in the teaching of embedded system in colleges and universities and suggested teaching reform methods for embedded systems in view of these problems. The experimental part evaluated the online drug ingredient recognition-embedded monitoring system and the embedded system teaching. The research results showed that the online drug ingredient recognition-embedded monitoring system had high ACC, SEN, SPE, and MCC, and the embedded system teaching gained high student satisfaction.
